# The use of patient experience survey data by out-of-hours primary care services: a qualitative interview study

**DOI:** 10.1136/bmjqs-2015-003963

**Published:** 2015-10-21

**Authors:** Heather E Barry, John L Campbell, Anthea Asprey, Suzanne H Richards

**Affiliations:** University of Exeter Medical School, University of Exeter, Exeter, Devon, UK

**Keywords:** Primary care, Qualitative research, Health services research, Patient satisfaction

## Abstract

**Background:**

English National Quality Requirements mandate out-of-hours primary care services to routinely audit patient experience, but do not state how it should be done.

**Objectives:**

We explored how providers collect patient feedback data and use it to inform service provision. We also explored staff views on the utility of out-of-hours questions from the English General Practice Patient Survey (GPPS).

**Methods:**

A qualitative study was conducted with 31 staff (comprising service managers, general practitioners and administrators) from 11 out-of-hours primary care providers in England, UK. Staff responsible for patient experience audits within their service were sampled and data collected via face-to-face semistructured interviews.

**Results:**

Although most providers regularly audited their patients’ experiences by using patient surveys, many participants expressed a strong preference for additional qualitative feedback. Staff provided examples of small changes to service delivery resulting from patient feedback, but service-wide changes were not instigated. Perceptions that patients lacked sufficient understanding of the urgent care system in which out-of-hours primary care services operate were common and a barrier to using feedback to enable change. Participants recognised the value of using patient experience feedback to benchmark services, but perceived weaknesses in the out-of-hours items from the GPPS led them to question the validity of using these data for benchmarking in its current form.

**Conclusions:**

The lack of clarity around how out-of-hours providers should audit patient experience hinders the utility of the National Quality Requirements. Although surveys were common, patient feedback data had only a limited role in service change. Data derived from the GPPS may be used to benchmark service providers, but refinement of the out-of-hours items is needed.

## Introduction

In England, out-of-hours primary care services provide urgent medical care to patients when their general practitioner (GP) surgeries are closed, typically between 18:30 and 8:00 on weekdays, and at weekends and bank holidays.^1^ Provision of out-of-hours primary medical care in England has changed significantly over the last decade. Most out-of-hours primary medical care is provided by a mix of National Health Service (NHS) organisations, not-for-profit social enterprises and commercial organisations, commissioned by local Clinical Commissioning Groups (CCGs); only a minority of GPs (10%) provide out-of-hours care for patients registered with their practice.^2^ The phased introduction of the NHS 111 service across England since 2013 has now altered how patients access out-of-hours services. Rather than contacting services directly, patients now telephone ‘111’ and their call is triaged before being signposted on to out-of-hours primary care services, where appropriate.

Within Western countries, there is variation in the models of delivery of out-of-hours primary medical care for patients with urgent healthcare needs, influenced by different healthcare systems.^3^ ^4^ Indeed, it is not uncommon for a mixture of models of delivery to coexist within single countries.^3^ Notwithstanding this, the delivery model of out-of-hours primary medical care in England is similar to that of other European (The Netherlands, Iceland, Ireland, Scotland, Sweden, Wales) and Southern Hemisphere nations (Australia, New Zealand), all of which have moved away from single-practitioner models to larger family-doctor based models (such as GP cooperatives and deputising services).^3^ ^4^ Some countries, such as Austria and the USA, still rely on around-the-clock care provided by individual GPs while in others (Canada, France), hospital emergency departments predominate.^3^

Urgent care provision in England has been criticised regarding service accessibility, the lack of continuity of care and concerns about patient safety.^5–10^ Similarly, there is great interest about quality and safety of out-of-hours care provision among researchers and policy makers internationally,^4^ ^11^ as there are risks and benefits to all the different models of delivery. A review of urgent and emergency care services in England is underway^12^ and the Care Quality Commission has assumed responsibility for regulating and inspecting out-of-hours services and is piloting a new inspection approach from October 2014.^13^ Providers are also expected to comply with National Quality Requirements (NQRs).^14^ NQR5 requires providers to regularly audit a random sample of patients’ experiences and to act on the results. From 2015 CCGs will be expected to publish annual data on provider performance against the NQRs.^1^ This is problematic for NQR5, as there is no agreed methodology on how providers should conduct patient audits (there is no recommendation as to which survey instrument to use), despite the availability of validated survey instruments,^15–18^ and hence benchmarking is not possible.

Patient experience of out-of-hours care is monitored through national surveys of several healthcare systems. For example, in the USA the Patient-Centred Medical Home Survey, which is part of the Consumer Assessment of Healthcare Providers and Systems Clinician & Group survey, contains two questions on out-of-hours care; whether the respondent has been given information on how to obtain care after hours, and whether they are given reminders between visits.^19^ The Australian Bureau of Statistics Patient Experience Questionnaire asks four questions regarding whether respondents have sought out-of-hours care in the previous year and whether they faced barriers to accessing care.^20^ In England, the national GP Patient Survey (GPPS)^21^ is distributed to a probability sample of 2.6 million patients, who are registered with a GP in England every year, irrespective of whether they have contacted their GP during that period.^22^ GPPS is part of the NHS policy initiative to improve patient experience and facilitate patient-centred care. Patients are invited to provide feedback about their experiences of local NHS services, including questions about access to GP services, interpersonal aspects of care, care planning and dentistry. There are six items relating to out-of-hours care (comprising two ‘access’ and four ‘evaluative’ questions).

As the first and only large-scale population survey of patients’ understanding, use and experiences of out-of-hours care in England, benchmarking of GPPS data is potentially possible. The National Audit Office recently used GPPS data to monitor patient satisfaction with GP out-of-hours services.^2^ However, these data were reported at CCG level precluding direct comparison between providers (who are often commissioned by two or more neighbouring CCGs). While monitoring and improvement of patient experience is considered of great importance within the NHS, it has been reported previously that the potential of patient experience surveys to impact upon change within primary care is limited.^23^ ^24^

This qualitative study investigates how out-of-hours providers routinely collect patient feedback, and how they use it to inform and shape service provision. Staff from out-of-hours service providers were also asked to reflect on the use of patient experience data from the out-of-hours items from the national GPPS.

## Methods

### Sampling and data collection

Six out-of-hours primary care service providers were already recruited and had taken part in a survey study conducted by the research team.^25^ We sought to recruit up to six additional providers (n=12 in total) in this study, although provider and staff recruitment would cease when data saturation was achieved. Providers were selected purposively from within groupings of interest. Providers were sampled primarily on the basis of their scores for the GPPS item rating for care received from the service (Question 40, April–September 2010 data) to achieve diversity of high, medium and low scoring services. Once providers were categorised into these groupings, additional information on the organisation type and geographical location was considered. Final selection of providers ensured diversity in range of score, type of organisation and geographical area. The research team aimed for diversity to ensure representativeness of providers, but from the outset no comparison of different subgroups of providers was planned. Up to three interview participants per provider were approached to take part in an interview. Potential interviewees were identified by a key contact within each provider organisation, on account of their involvement in conducting patient experience surveys, and included those who had administrative, managerial and clinical duties within the organisation. Participants were approached by the research team and provided with an information pack comprising a covering letter and a participant information sheet. HEB arranged to conduct the interview at a mutually convenient time.

A ‘feedback report’ was provided to each participant at least 1 week before the interview. The report contained patient ratings of their out-of-hours service derived during the July 2012–March 2013 wave of the GPPS. Benchmarking data were provided to allow providers to compare their performance to that of the 91 other English out-of-hours services for whom we were able to generate scores. Benchmarking data were generated by matching GP practice postcodes to providers’ localities. For services that had participated in the survey study (n=6), reports also summarised the provider's ratings derived from the research survey (see online supplementary appendix 1).

Qualitative data were collected by HEB via face-to-face interviews with participants. Interviews, which were usually conducted at the participant's workplace, took place between April and July 2014. Written informed consent was obtained before commencing the interview. Interviews lasted between 39 min and 88 min (mean: 59 min). Each semistructured interview used a topic guide whose content was developed from a review of the literature, from discussion between the researchers and service providers, and from findings of previous research (see online supplementary appendix 2). A study advisory group, comprising primary care academics, representatives from out-of-hours services and a service user, commented on the content of the topic guide. The topic guide included questions on: how providers routinely collect patient experience data and how it is used to make changes to service provision; participants’ awareness of the GPPS, their views on the out-of-hours items in it, and reflections on the utility of the GPPS benchmarking data provided within their feedback report.

### Analysis

All interviews were digitally recorded and transcribed verbatim. Each transcript was checked against the original digital recording for accuracy and anonymised to ensure participants could not be identified. Transcribed data were entered into NVivo V.10 software (QSR International, 2012). All analysis was carried out by the principal analyst (HEB) in an iterative process with an additional analyst (AA) independently coding the first five transcripts to ensure agreement was reached on the emergent coding frame and themes. A rudimentary ‘framework’ was constructed to reflect the topic guide,^26^ and use of the constant comparative method^27^ ensured that any new themes arising from the data collected were identified and added to the amended coding frame. These codes were tested through seeking negative cases and/or divergent data, and the data were reorganised and collapsed into overarching themes, until the main categories were agreed through discussion with all authors. Interview findings were combined into a summary that was sent to all participants with a structured feedback form inviting comments on the veracity of the interpretation of the study findings. Final themes were reviewed and agreed between HEB, AA, JLC and SHR to enhance reliability.

## Results

Of six services approached (in addition to the six who participated in the original survey work) five agreed to take part. No reason was provided by the service that declined to participate. From the 11 participating out-of-hours providers (n=2 NHS organisations, n=4 social enterprises, n=5 commercial organisations) 31 staff agreed to be interviewed, at which point data saturation was judged to have been reached. Most participants were female (n=23); 18 were out-of-hours service managers, 7 were clinicians (GPs) and 6 were administrators.

Two participants completed a feedback form commenting on the study's findings, and both were satisfied that the summary accurately reflected their views and experiences that were expressed during the interview. Therefore no amendments were made to the summary and the authors’ interpretation of the interview data remained unchanged.

Three main themes emerged: using surveys as a method of obtaining patient feedback; the utility of patient feedback; and the value of benchmarking. Each theme is illustrated through the use of quotations. Pseudonyms have been used where necessary to protect anonymity, and hesitations and repeated words have been removed from quotes to enhance readability.

### Surveys as the most common method of obtaining patient feedback

Interviewees described how their service obtained patient feedback and reflected on this process. Most participants focused on survey methods, as 10 of the 11 providers undertook regular surveys to audit their patients’ experiences. Participants also discussed the ambiguities of operationalising NQR5, the desire for qualitative feedback to supplement survey data, and the role of alternative methods in addition to surveys.

#### Ambiguities in operationalising NQR5

Through describing how services administered patient surveys, it was evident that each provider interpreted NQR5 differently, particularly with regards to patient sampling. Each provider was auditing a different proportion of patients, ranging from 1% through to 20%:

We send out approximately 250 a week. Our National Quality Requirements require us to survey 1%—we actually do considerably more than that because we have taken our own interpretation on it. We have discovered that there are some providers who think that 1% means you survey 1%, so if you see 100 people you survey 1, whereas we have interpreted that as we want to make sure we obtain feedback from 1%, so we actually survey a lot more to obtain that 1% feedback. A lot of people have said that that is not what it means, it doesn't matter to us, we thought that that was a good figure, so we send out more. I think it is 4% that we survey. (11_4001, Manager)

Participants reported that audits were undertaken on either a weekly or monthly basis, using survey instruments developed by their organisation. Services who administered patient experience surveys on a weekly basis found that this helped to maximise patient response rate:

They [the out-of-hours service] try to do it as contemporaneously as they can, but clearly that can be challenging, but I think because they've worked out that the sooner the patient gets the questionnaire the more likely it is that they will complete it because it's still fresh in their minds, so they try to do it as quickly as possible. (14_4003, GP)

#### Preference for qualitative feedback

Most participants placed great importance on qualitative feedback from free-text comments recorded by patients in surveys. Interviewees explained that these comments helped them interpret the quantitative ratings. More importantly perhaps, this richer detail was felt to identify areas where action might be taken:

If they have got a real issue they can put it down, can't they? Just doing the survey itself is just a way you test the water. How your survey is going and as we run it… it has gone all right, but we are not using it, it is pretty meaningless. The free-text allows someone who has got a very bad experience the opportunity to write to us. (10_4001, Manager)

A small number of interviewees expressed frustration that when asked to provide qualitative feedback, service users occasionally took the opportunity to comment on any aspect of the NHS, not necessarily the out-of-hours service:

You do get some obscure comments coming through but we're trying to whittle that sort of thing out. (15_4001, Manager)

On the whole, interviewees felt that qualitative feedback provided them with a more personalised response from their patients:

I'm dealing with people, I'm not dealing with robots. I mean, it's their experiences, their feelings and they need to have a place to feed that back. So just getting them to tick the boxes is not going to encompass the range of reactions that they may or may not have in relation to the service that they've received, and they absolutely need to have a place to express their opinions—that's giving people a voice. (14_4003, GP)

Some people do respond very specifically. So, in the situation where they've said, ‘Dr. So-and-so was whatever’, then that's great because we can lift it and we can give it back to that doctor. If it's negative then we want to know because we can then do something about it. (12_4003, Manager)

#### Alternative methods of feedback

Although patient surveys were deemed necessary by virtually all the providers, they were not deemed a sufficient resource in their own right as a facilitator of change. Participants reported using a wide variety of alternative methods to obtain patient feedback. Comment cards were used by a number of service providers, and ‘complaint and compliment’ systems were another valuable way of acquiring patient feedback. Some interviewees explained how new technologies were being explored:

At the moment we're thinking of going more electronically, so as soon as you have your consultation in the base, you come out and there's a tablet so you can actually do your surveys straight after, in which case the names of the doctor and receptionist are fresh in your mind—that way you can get more accurate feedback of how people are feeling. (19_4002, Administrator)

Although much less common, some organisations reported also collating patient feedback through patient interviews, from local GP surgeries, mystery shopper initiatives, patient groups and via staff members.

### Utility of patient feedback

Interviewees focused on how patient feedback was used to inform changes to out-of-hours service provision. Participants also reflected on barriers and facilitators to bringing about change within their organisations.

#### Making changes to service provision

Many participants cited examples of ways in which patient's reported experiences had been used to make changes to service provision. Most changes tended to be ‘low-level’, that is, things that were easily fixed, such as signage and the use of badges for identification of staff. Changes arising from patient comments regarding waiting times were common:

We know that one of the areas that we're working hard on in the operation team is that when they get to the treatment centre our lowest response rate is about knowing about waiting times and we're aware of that through the patient experience surveys. Consequently we are working hard to try and improve that, so we've put little flip things up saying ‘Your waiting times an hour’ and we're doing a lot of work trying to get the reception staff to be much more proactive rather than just ‘Oh, come in and take a seat’. One hopes that over a period of time you'll see improvements in that as the message gets home. (11_4003, Manager)

Most participants reported that patient survey data was insufficient to instigate service-wide changes due to the lack of clear trends observed within it:

In the main the results are stable and pretty good, but there's not enough that's consistent that I think we could use around wholesale service change. (12_4003, Manager)

The responses were few and we couldn't really do anything with them, they were meaningless because they were either so isolated, they didn't really form patterns. (13_4003, GP)

We obviously focus around feedback that isn't positive because we want to make sure we do something around service change. A lot of it is stuff that is individual to somebody, and there aren't significant themes you can take away from it that would drive a system-wide change in behaviour. A lot of it may be down to an individual clinician that was seen that day or it was a busy shift and that's generated a negative response. (12_4003, Manager)

#### Perceived barriers

A range of barriers to using patient feedback to enable change were identified. Many reported that patients’ expectations of the out-of-hours service were often unrealistic and difficult to manage, and this made patient feedback difficult to deal with:

You often get patients who are very unhappy about the service they got and when you drill down into it it's because they didn't get antibiotics for their cold. Its expectations. And I think one of the problems I have with surveys is, is it based on a reasoned, objective look at what you do or what's been done, or is it based on a subjective gap between expectations and the reality? (16_4003, GP)

Interviewees also identified those underlying, but fundamental changes to the landscape of the English urgent care system to be very confusing for patients. This led them to question the validity of patient feedback as the patients may not be aware that different elements of the care pathway may be provided by different organisations, that is, a ‘111 call handler’ versus an out-of-hours provider:

I think patients are very confused about the healthcare economy. When I get a complaint in, they will complain about a service and when you investigate, it's the 111 service, or 111 will get a complaint and it will be the out-of-hours service, or they'll complain and it will be Accident & Emergency. (20_4002, GP)

Another barrier was the low level of engagement by commissioners (the CCGs) reported by some out-of-hours staff; although patient experience audits are part of the NQRs, many staff reported that the CCGs treated it as a ‘tick box exercise’.

I think from a commissioner's perspective they're so busy with other things that there's a risk of it being lower down the priority order, and if we are evidencing to them our results and our partnership working out in the local communities with patients, then it probably ticks the right box for them and they don't look any more deeply into that. (12_4003, Manager)

They [the Clinical Commissioning Group] don't come across to me as particularly engaged in this at all, and never really ask us too many questions around it. They don't seem to be demonstrating to me that they are really actively that bothered with the responses that they're getting. That's the impression I get anyway. (18_4003, Manager)

#### Perceived facilitators

While interviewees tended to focus more on the barriers to change arising from patient feedback, some facilitators of change were identified. Interviewees discussed how engaging in patient feedback had subtly changed the culture within their organisation. Many reported it was important for services to be responsive to change and transparent about the patient feedback they received:

I guess it comes down to the drive and the desire of each individual provider as to how much they want to do that and how much they're willing to change as a result of doing that, the responses they get. I think we've been quite good and quite flexible to say ‘Well, let's listen to what our patients are saying and let's try and change it.’ I know you get the odd comment that you think ‘Well, we're never going to change, we're never going to do that differently.’ But there are a number of things that we have done and we have listened to patients. (18_4003, Manager)

Participants reported that being able to compare their patient feedback with other NQR measures (mainly process data, such as service response times) was beneficial in understanding patient experiences:

The National Quality Requirements are obviously another quality marker, so are we meeting the performance in terms of seeing people, key performance indicators, and then patient satisfaction is another layer of that feedback that if you are a commissioner you would want to know that the patient satisfaction is good, because good quality is not only safe, effective, but it is also good patient experience, so it is a key part of your determination of quality isn't it? (17_4003, GP)

### Value of benchmarking

Interviewees discussed the perceived value of benchmarking and comparative data for providers, and how the GPPS could potentially be used to provide benchmarking data for English out-of-hours services. Most participants acknowledged the benefits of having access to benchmarking data and felt this was a facilitator to enabling change.

Having benchmarking is important because you don't know what ‘good’ is unless you have got a benchmark to start with. And if you've got somebody who you can see is a good provider, you think ‘Well what are they doing that their patients are much happier?’ So therefore I can learn from them. (11_4003, Manager)

However, some interviewees expressed concerns that providers were reluctant to share with and learn from others, an issue mainly arising (as some perceived it) from the commercialisation taking place within the NHS:

The problem with out-of-hours is that in theory it's a contract that's up for retendering and people are in competition and so sharing best practice doesn't happen. I think people have their trusted friends they share practice with, but actually to sort of share it with the whole community, you wouldn't want to share with someone who's on the border and who might be a competitor to bid for a service. (12_4001, Manager)

It's terrible isn't it, when everybody's competing and not collaborating? That's the system we're living with, we've had to get used to it. (18_4001, GP)

When reflecting specifically on the GPPS out-of-hours patient ratings included in the feedback report, interviewees generally found the benchmarking useful:

Yes, it was useful, it was reassuring to know how we are doing in comparison to the national average, and again it is that confirmation of what you are doing well, and what you should keep doing, especially when you are in a world of budget cuts and retendering. (11_4001, Manager)

However, many interviewees identified weaknesses with the GPPS out-of-hours items including reports that the questions were not reflective of the current urgent care system, lacked detail and could be ambiguous to respondents:

*It is* [General Practice Patient Survey out-of-hours evaluative items] *just four questions, you get asked in McDonalds. It's not detail is it?* (10_4001, Manager)

What is confidence and trust? I don't understand what that means. So again, it goes back to what I was saying, what are these questions, what are they asking, what are the responses they want and is a tick box the right way to have such an important thing measured? (16_4003, GP)

While many were interested in its benchmarking potential, most interviewees did not feel that their GPPS data would drive service change. Indeed, they placed greater importance on their own patient surveys:

I think it should be discussed, circulated to everybody in our staff and we should have a meeting to discuss this report and see whether in fact some people do feel that there are things that we alter or modify. I do believe that we should have discussions about things; we don't put things under the carpet so they feel uncomfortable. I think one should look to see if there is any validity in what is actually being said here or not. (13_4003, GP)

I've acknowledged it and I found it valuable comparatively, but I'm not jumping up and down. I don't know how it's going to add value to what we already have in the pipeline. (20_4002, GP)

## Discussion

In the UK out-of-hours primary care providers are mandated to regularly audit patients’ experiences as part of the NQRs. We found that services routinely met this requirement by conducting patient surveys, as well as by obtaining feedback using a variety of other methods. It was clear, however, that NQR5 is ambiguous and that the resultant data cannot be used to compare services; providers are undertaking audits of varying scale and frequency, using a variety of survey tools of uncertain and/or variable psychometric properties. Our participants reported a strong preference for qualitative patient feedback. Echoing research undertaken in other settings, a range of methods were used, all of which had the potential to yield richer, more detailed feedback than quantitative survey scores. For example, hospital staff have found qualitative feedback from their patients more useful than survey scores, and felt that qualitative data added a more patient-centred aspect to patient satisfaction measurements.^28^ ^29^ A study of healthcare leaders found that they placed great importance on complaints, comments and compliments as a source of patient feedback,^30^ as did GP practice staff.^31^

Our participants reported that patient feedback had a limited role as a driver for service change. Moreover, effective change is hindered by modifications taking place in the urgent care landscape. Staff reported that many patients seem to lack an understanding of how care was organised, and some patients had unrealistic expectations of what out-of-hours services could deliver. Some staff also reported that commissioners appeared uninterested in patient experience audit findings. Audit and feedback has been shown to have small to moderate effects on healthcare professionals’ practice,^32^ ^33^ although in some settings it can have a wider impact.^34^ In particular, the organisational culture must be supportive of change and be patient-focused for change to ensue.^35–37^ While staff tasked to act on patient feedback had a clear desire to make their services more responsive, a key challenge relates to data quality. Most of the changes that were cited were ‘low-level’ and unlikely to drive system-wide reconfiguration due to the lack of consistent patterns observed in the data. The preference for qualitative feedback appeared driven by the fact that patient free-text comments have the potential to identify specific areas of actionable change, or through contributing to wider data-gathering audits (eg, critical incident techniques).^38^ However, in order to be useful, patients’ attention must be focused to provide qualitative feedback on the out-of-hours service.

Staff valued the GPPS patient experience benchmarking data. Some had participated in benchmarking exercises conducted by participants in Urgent Health UK (a federation of social enterprise out-of-hours primary care providers) and the Primary Care Foundation.^39^ However, these activities are only available to members (Urgent Health UK) or to services electing to join the exercise (Primary Care Foundation). The GPPS presents an opportunity for benchmarking of all out-of-hours services. NHS England has recently recommended that CCGs use the GPPS results to monitor patient experiences with out-of-hours providers,^1^ and the Care Quality Commission has published GPPS provider performance at CCG level.^13^ Major strengths of the GPPS are that it is conducted on a regular basis, run by an independent organisation (Ipsos MORI), with results made publicly available. However, we found participants were reluctant to use GPPS data in its present form due to concerns about face validity of out-of-hours items and the absence of free-text comments, which previous studies also found to be a limitation.^31^ ^40^ In addition, the out-of-hours items are not currently reflective of the recent changes that have taken place within the urgent care system, particularly access to out-of-hours services now via the NHS 111 service (often a different provider). Most staff did not believe that the limited number of GPPS items would drive change by themselves.

### Strengths and limitations

We investigated current practice within out-of-hours primary care providers, and examined the views of staff who have an in-depth knowledge of patient feedback processes within their organisation. To the best of our knowledge, this is the first qualitative study to explore these issues. Our sampling ensured that staff from a variety of different types of providers (eg, not-for-profit or commercial enterprises) serving diverse populations across England was sampled. We did not encounter major difficulties during recruitment, and found that nearly all of the service providers we approached agreed to take part. Although we achieved sampling diversity, we acknowledge that participating organisations may be more interested in the patient experience agenda than non-participants, and thus our findings may not reflect the views of the wider population. The views of commissioners were not sought in this study. That we found a widespread perception that some commissioners were apathetic towards patient feedback data must be interpreted cautiously. Due to logistical constraints we were unable to interview commissioners and obtain their perspective on their perceived role and value.

### Implications and future research

Improving patient experience remains a major NHS policy issue in the UK.^41^ ^42^ Ascertaining patients’ experiences, alongside measures of clinical effectiveness and patient safety, are important indicators of quality of care.^43^ Although the NQRs are intended to promote transparency and allow comparisons between out-of-hours providers, we found that NQR5 was ambiguous and in its current form does not support benchmarking or service improvement. Our data revealed the tensions felt by service providers who desire benchmarking data, but cannot collect the detail required to provide this. Indeed, there is a strong desire internationally for benchmarking data in order to monitor quality.^44^ A critical review of the NQRs is needed in order to help providers to engage with patient feedback and drive service improvement effectively. In the absence of clear NQR guidance, we found providers to be creative in the ways in which they engage with patients.

National surveys, such as those used in England, the USA and Australia, provide a vehicle upon which patient experience of out-of-hours care could be monitored and benchmarked. However our data suggest that all three national surveys lack the granular detail of patient experiences of out-of-hours primary medical care required to enact service improvement. We found that patient qualitative feedback was highly valued as it provided detailed information which could lead to actionable changes, often when combined with more detailed investigation and process data. Despite this desire for qualitative feedback, free-text questions can be difficult to incorporate into national surveys and the resulting data can prove time-consuming to process, and can be vague and difficult to make sense of.^45^ Services were struggling to find ways to use patient feedback to drive anything other than low-level service change, similar to other studies which have reported that patient feedback often fails to result in improvements in service delivery.^45^

Future research should ascertain patients’ views on the feedback data from GPPS out-of-hours items in the UK. In addition, while much is known about how to collect patient feedback,^46^ and of the wider organisational culture that supports quality improvement approaches,^47^ ^48^ our study emphasised the need for future work to focus on the ‘translational gap’. Future research is needed to explore how out-of-hours services can be assisted in engaging more fully with patient feedback, and whether comprehensive guidance on how to collect, interpret and act upon patient feedback has the potential to drive quality improvement initiatives (irrespective of the healthcare system or model of service delivery).^23^ ^36^ ^37^ However, within the context of the rapidly changing landscape of UK urgent care services, while participating services could see the potential of using GPPS for benchmarking purposes, its out-of-hours items need urgent revision as they do not reflect current telephone access arrangements for out-of-hours care.

## Conclusions

This study highlighted the uncertainties that out-of-hours providers face when gathering and acting upon patient feedback, which are relevant to other healthcare systems and models of service delivery. Patient feedback currently has a limited role in driving changes to service provision, and the utility of feedback may be hindered, in part, by recent modifications to the UK urgent care system and lack of clarity of NQR standards relating to gathering and acting upon patient feedback. Providers valued benchmarking data derived from the national survey and its ability to compare service providers. However, such information does not replace the need for more granular information collected by services using a range of different methods. The GPPS out-of-hours items also need to be updated to reflect the changes made to accessing out-of-hours services by telephone, so that providers can be confident that ratings reflect their services’ performance.

## Supplementary Material

Web appendix1

Web appendix2
