# Supplementary material for: The use of patient experience survey data by out-of-hours primary care services: a qualitative interview study
Source: BMJ Qual Saf. 2015 Oct 21;25(11):851–9. doi: 10.1136/bmjqs-2015-003963 (PMC5136714; doi:10.1136/bmjqs-2015-003963)
Supplement: Web appendix1 [file bmjqs-2015-003963-s1.pdf]

Supplementary file, Appendix 1 – Feedback report

Structure of the report

The feedback report began with a short introductory section. This provided a background to the GP Patient Survey (GPPS) and the purpose of the present study. An explanation was given as to how the national survey data were manipulated in order to provide the benchmarking data, and how these data were presented within the report. Contact details for the research team were provided, should participants have required further information or clarification about the content of the report.

For the service providers who had participated in the earlier survey study (*n*=6), their data from this study were presented in the form of basic frequencies and percentages. All of the data were tabulated and included in an appendix to the report. Any free-text responses which were received from survey respondents (which were anonymised where necessary) were also included in an appendix to the report.

Comparative data, collected routinely by the national GPPS, were presented for all participating service providers (*n*=12) for the four evaluative questions: (1) How easy was it to contact the GP out-of-hours service by telephone? (2) How do you feel about how quickly you received care from the out-of-hours GP service? (3) Did you have confidence and trust in the out-of-hours clinician you saw or spoke to? (4) Overall, how would you rate your experience of out-of-hours GP services? These data were collected from surveys distributed during the period July 2012 – March 2013.

Generation and presentation of benchmarking data

The research team 'mapped' GP practices to the areas covered by out-of-hours services to generate a provider score (i.e. by combining service users' ratings from these practices). A list of practices located within different out-of-hours provider areas was produced by the information team from NHS England (November 2013).

An overall **adjusted score** was produced for each of the four evaluative GPPS questions. This was adjusted for demographic variables (age, gender, ethnicity and deprivation data) and converted to a scale of 0-100 (where 100 was the highest possible score and 0 the lowest possible score). A graph was produced for each evaluative question showing how the service provider's overall adjusted score for each item compared to the overall adjusted score for all 92 out-of-hours service providers in England.

Each service provider was also informed as to which **quintile** their score fell into, represented pictorially beside the bar chat (see Figure 1). Quintiles were generated by sorted the overall adjusted scores from the 92 English providers into rank order, then allocating each provider's score into one of

five equal categories. Quintile 1 represented the providers rated most highly by service users (i.e. highest 20%) while quintile 5 represented the lowest scoring service (i.e. lowest 20%).

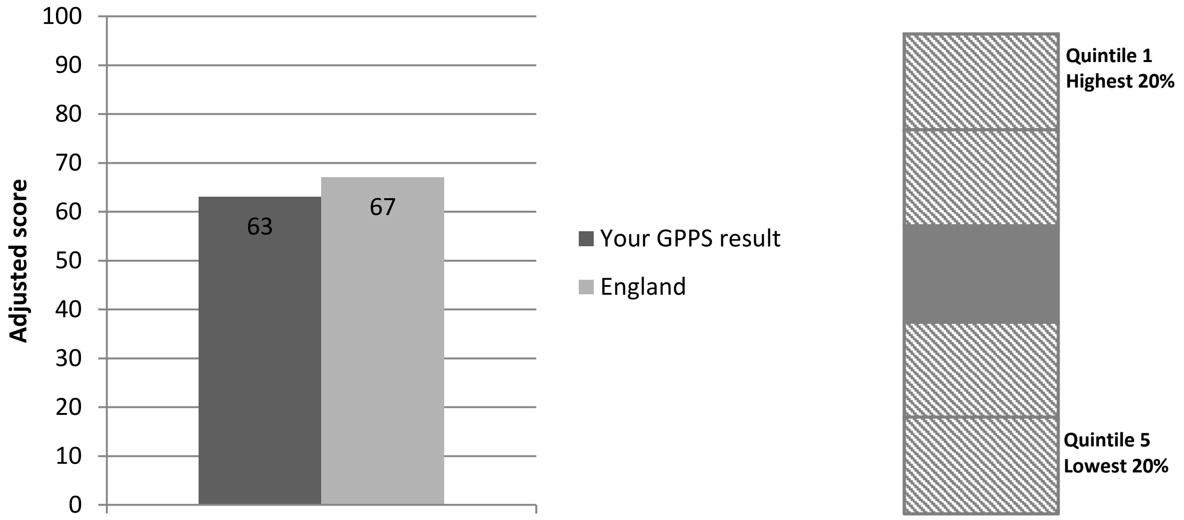

Figure 1. Overall, how would you describe your experience of out-of-hours GP services?

Challenges

Due to major ambiguities in the list supplied by NHS England, it was not always possible to conclusively map each GP practice to just one out-of-hours service provider. We adopted a cautious approach, excluding such practices from the dataset rather than just allocating their patient ratings to two or more providers. Service providers were informed within the feedback report as to how many local GP practices their information was based upon. Providers were advised to exercise when interpreting the data, as it may not have represented the full diversity of service users in their catchment area.
