# Supplementary material for: The use of patient experience survey data by out-of-hours primary care services: a qualitative interview study
Source: BMJ Qual Saf. 2015 Oct 21;25(11):851–9. doi: 10.1136/bmjqs-2015-003963 (PMC5136714; doi:10.1136/bmjqs-2015-003963)
Supplement: Web appendix2 [file bmjqs-2015-003963-s2.pdf]

Supplementary file, Appendix 2 – Interview topic guide

Patient surveys of out-of-hours primary medical care

Please describe briefly how your organisation routinely surveys patient experience for the purposes of satisfying National Quality requirements.

What are the key challenges in getting data from your survey population?

What mechanisms for change are in place in your organisation to channel and act upon this feedback?

In your opinion, what is the role of the surveys of patient experience in moving towards standardisation of out-of-hours?

Can you tell me the ways in which your organisation has responded to the results of these surveys?

What needs to be done to make better use of surveys and to achieve improvements at the level of local and national decision-makers?

In your opinion, are there any other ways to receive patient feedback needed for improvements?

How do service users know they have been listened to, and their suggestions have been implemented?

The national GP Patient Survey and feedback report data

What do you know about the GP Patient Survey in general? Does your service use information from it?

Do you see any advantages or disadvantages of a national survey such as the GPPS?

How well, in your view, do the questions on out-of-hours care reflect patients' experiences of out-of-hours primary medical care?

Did you find the benchmarking data within the feedback report useful? How did you interpret it?

Is the level of information captured suitable for use in your National Quality Standards reporting? If not, what is missing?

Based on your GPPS results, did you make or propose any changes to your service to improve patient experience?

GPPS doesn't capture free text – do you feel this is/would be useful? What do you feel is the role of free-text responses in surveys?
